# Working at the interface in Aboriginal and Torres Strait Islander health: focussing on the individual health professional and their organisation as a means to address health equity

**DOI:** 10.1186/s12939-016-0476-8

**Published:** 2016-11-17

**Authors:** Annabelle M. Wilson, Janet Kelly, Anthea Magarey, Michelle Jones, Tamara Mackean

**Affiliations:** 1 Southgate Institute for Health, Society and Equity, Flinders University of South Australia, GPO Box 2100, Adelaide, South Australia 5001 Australia; 2 School of Nursing, University of Adelaide, North Terrace, Adelaide, Australia; 3 Nutrition and Dietetics, Flinders University of South Australia, GPO Box 2100, Adelaide, South Australia 5001 Australia; 4 Adjunct Research Fellow, University of South Australia, Adelaide, South Australia 5000 Australia

**Keywords:** Aboriginal and Torres Strait Islander, Indigenous, Interface, Practice, Health professional

## Abstract

**Background:**

Aboriginal and Torres Strait Islander people experience inequity in health outcomes in Australia. Health care interactions are an important starting place to seek to address this inequity. The majority of health professionals in Australia do not identify as Aboriginal and/or Torres Strait Islander people and the health care interaction therefore becomes an example of working in an intercultural space (or interface). It is therefore critical to consider how health professionals may maximise the positive impact within the health care interaction by skilfully working at the interface.

**Methods:**

Thirty-five health professionals working in South Australia were interviewed about their experiences working with Aboriginal people. Recruitment was through purposive sampling. The research was guided by the National Health and Medical Research Council Values and Ethics for undertaking research with Aboriginal communities. Critical social research was used to analyse data.

**Results:**

Interviews revealed two main types of factors influencing the experience of non-Aboriginal health professionals working with Aboriginal people at the interface: the organisation and the individual. Within these two factors, a number of sub-factors were found to be important including organisational culture, organisational support, accessibility of health services and responding to expectations of the wider health system (organisation) and personal ideology and awareness of colonisation (individual).

**Conclusions:**

A health professional’s practice at the interface cannot be considered in isolation from individual and organisational contexts. It is critical to consider how the organisational and individual factors identified in this research will be addressed in health professional training and practice, in order to maximise the ability of health professionals to work with Aboriginal and Torres Strait Islander people and therefore contribute to addressing health equity.

## Background

Inequity in the health of Aboriginal and Torres Strait Islander^1^ people is well documented in Australia [1]. For example, Aboriginal Australians experience a burden of disease two and a half times greater than non-Aboriginal Australians [1], and Aboriginal people born between 2010 and 2012 are estimated to have a life expectancy that is between 9.5 (males) and 10.6 (females) years lower than for non-Aboriginal Australians [2]. Factors contributing to such inequity include processes of colonisation, discriminatory policies and practices and racism [3, 4]. The social determinants of Indigenous health clearly demonstrate that the health of Aboriginal Australians is affected by multiple factors [5].

It is well known that provision of health care to Aboriginal people by Aboriginal people improves access to appropriate health care and subsequently improves health outcomes and addresses inequity in health [6, 7]. An increase in Aboriginal staff within the health sector is important and needs to be continued and amplified into the future [8]. While efforts have been made to increase the number of health professionals who identify as Aboriginal and/or Torres Strait Islander, in Australia, only one percent of the health workforce identify as Aboriginal and/or Torres Strait Islander [9]. Hence the health care interaction with Aboriginal clients/patients in Australia will usually involve a non-Aboriginal health professional and an Aboriginal client/patient and this is therefore within an ‘intercultural space’. The intercultural space has been variously described. Willis et al. [10] present the notion of a third space in Aboriginal healthcare, Ermine [11] (p. 193) describes the ‘ethical space of engagement’, which is ‘formed when two societies with disparate worldviews are poised to engage each other’ and Durie [12] describes the ‘interface’ where two different knowledge systems come together and new knowledge is created that can be used to advance understanding in both worlds. Working effectively in the intercultural space is crucial if health professionals, health students and health educators are going to contribute to closing the gap; studies have shown that it is in this intercultural space that actual work and true collaboration are achieved [10, 13]. However, the intercultural space is not necessarily a safe space for either patient or health professional [13]; disquiet, discomfort and/or anxiety, often arise as culture, identity and Indigenous health issues are experienced individually, and often differently, within this space [14],

There has been little investigation into the factors that influence a health professional’s experience of working in this intercultural space, henceforth referred to as ‘the interface’ in this paper. A well-trained workforce is required to address Aboriginal health concerns [15] and there is a need to assist health professionals to come to terms with the difficulties, discomforts and emotions experienced in cross-cultural contexts [16]. It is therefore necessary to identify the specific factors that influence a health professional’s experiences of working at the interface so that these factors can be addressed through health professional training and professional development. This will support maximising the benefit of the healthcare interaction and thus address health inequity.

The purpose of this paper is to identify what factors influence the experience of health professionals working at the interface.

## Methods

### Ethics approval

Ethics approval for this study was granted by the Flinders University Social and Behavioural Research Ethics Committee, the SA Health Human Research Ethics Committee, the South Australian Aboriginal Health Research Ethics Committee and the ethics committee of the Department of Education and Children’s Services.

### Researcher

The primary researcher (Annabelle Wilson) identifies as a white dietitian and researcher. She has worked with Aboriginal communities across Australia during the past 10 years. Her research and practice is strongly guided by learnings she has obtained from working in partnership with Aboriginal individuals and communities.

### Theoretical framework and guiding principles

The research was guided by the National Health and Medical Research Council (NHMRC) Values and Ethics for undertaking research in partnership with Aboriginal communities [17], in particular the principle of reciprocity. These principles were upheld by the primary researcher through activities including attending local community events, working with Aboriginal project mentors, attending community lunches, consulting with Elders’ Committees and activities of reciprocity (giving back) based on what was requested by the community. Over the duration of the research project, the researcher spent between half and 1 day per week in activities of reciprocity with community members.

This study used a social constructionist epistemology [18, 19], which understands reality as experienced, or constructed, by the individual [20]. This approach was used to capture the different views of health professionals about their work in Aboriginal health and to recognise that individual experience shapes this understanding. Two main methodologies were used; critical social research [21] and reflexivity [22]. Critical social research guided data analysis (see below) while reflexivity guided the entire study. In particular, reflexivity was used to assess attitudes and potential biases of the researcher [22] and these were carefully examined through a critical reflexive journal. The results of this critical self-analysis have been published elsewhere [23].

### Recruitment, setting and sample

This study was conducted in South Australia in 2010 and allied health professionals were invited to participate in an interview. Sampling was by convenience and participants were selected purposefully based on their experience and/or interest of working in Aboriginal health. Purposive sampling ensures that participants have relevant experiences to contribute [24] and are information rich based on these experiences [25]. Allied health professionals were approached face to face and through email, primarily through two local health services where the researcher was based. Allied health professionals were the main staff employed at these two health services and hence they were chosen as the primary group to interview due to convenience. Health professionals included dietitians, occupational therapists, health promotion workers and speech pathologists, some of whom were working in a role as an allied health professional, and others who were working in a project coordination or management role. Dietitians were also recruited through a presentation at a state nutrition meeting and an email flyer sent to the group, of which the researcher was a part. The inclusion of extra dietitians was due to the professional background of the researcher.

### Data collection

Written consent was received from participants before each interview. The researcher undertook the interviews at a time and location convenient for the participants. Data were collected through semi-structured interviews that were digitally recorded and varied from 20 to 90 min in length. Interview schedules were designed to stimulate conversation about working in Aboriginal health. The interview schedule was developed by the primary researcher and reviewed by other authors. It was also reviewed by Aboriginal project mentors prior to use. This was crucial to obtain the input of Aboriginal people including worldviews and experiences into the data collection process. Examples of questions from the interview schedule have been included (Table 1). These questions have also been published elsewhere [26].

**Table 1:** Example interview questions

1	I would like to talk now about your role as …… at …….., and any experiences you have had with the Aboriginal community through that role.
2	What do you see your role, as …………, to entail in terms of working with the Aboriginal community?
3	During your time as ……….., did you attempt to work with the Aboriginal community? If no – why not? If yes – explore: process of engagement/contact, projects worked on, outcomes and barriers/enablers
4	What learnings have come out of your work with the Aboriginal community?
5	What do non-Aboriginal people need to know when working with Aboriginal people?
6	How do you demonstrate a commitment to Aboriginal health through your work?
7	As a non-Aboriginal person working ……….., what stops you or helps you to work with the Aboriginal community?
8	What are some of the beliefs that non-Aboriginal people hold about working with Aboriginal communities? How do you think that this impacts on their work?
9	Do you think that colonisation still impacts on the lives of Aboriginal people? Would you address this in the way that you work with Aboriginal people?
10	Do you have any other comments or is there something you thought I would ask that I have not?
11	In your experience are new graduate health professionals equipped to work with Aboriginal people? Why or why not?

### Data analysis

Interviews were transcribed verbatim and transcripts were de-identified and imported into QSR NVivo 8.0 software (QSR International, Doncaster, Victoria, 2008). Data were coded into themes that recurred throughout the interview by the primary researcher and these were reviewed by supervisors with professional knowledge and experiences in the field. Critical social research was used as a tool for further analysis to guide emergence and evolution of themes [21]. This was achieved using the process of deconstruction and reconstruction [21]. This process enables the researcher to expose the data by breaking it down into its individual elements (that is, attitudes and characteristics of participants) and then putting it back together in a different way to expose deeper meaning. Approximately half the participants took the option to review their own transcript and make changes. Of these approximately half made minor changes to the transcript (for example changes to sentence structure and meaning) and in these cases, the revised transcript was used by the researcher for data analysis.

## Results

A total of 35 health professionals participated. All identified as non-Aboriginal. Thirty two were female and 21 were from a rural location. The length of time participants had worked in Aboriginal health varied and included 0–1 year (*n* = 8), 1–5 years (*n* = 13), 5–15 years (*n* = 7) and more than 15 years (*n* = 7). Interviewees included dietitians (*n* = 21) and health professionals working in management and project management roles (*n* = 14). These professionals had a background in speech pathology, occupational therapy, health promotion, men’s health and/or women’s health but were not necessarily directly practising in their background area at the time of interview.

**Fig. 1:**
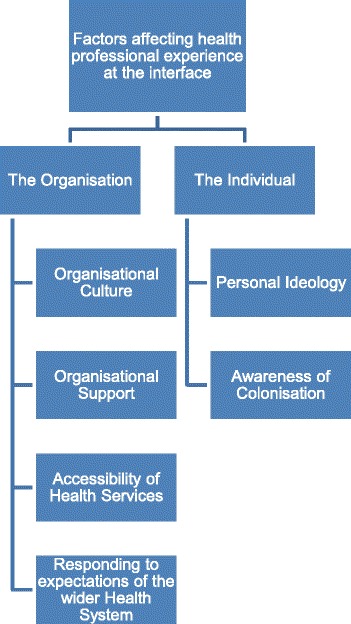
Factors influencing the experience of health professionals working with Aboriginal people at the interface

Two main types of factors influencing the practice/experience of non-Aboriginal health professionals with Aboriginal people at the interface: the organisation and the individual (Fig. 1) emerged.

### The organisation

Four key areas were identified within organisations that affected the experience of health professionals working at the interface. Within each of these four areas, elements of the organisation were found to either enable this interaction, or make it more challenging.

### Organisational culture

Non-Aboriginal health professionals felt enabled to work with Aboriginal people when their organisation had an ***ethos or an expectation*** that they would do this. Having spaces or groups where Aboriginal and non-Aboriginal people could come together, work together and share stories promoted development of partnerships. In one community, a ‘working together’ group paved the way for non-Aboriginal health professionals to begin and continue discussions with the Aboriginal health team.

Interviewees highlighted the importance of *cultural training*, delivered at the organisational level, to help create awareness and challenge some of the stereotypes and bias about Aboriginal people that participants had previously been exposed to. The importance of this training was described in detail by one worker:

…*non*-*Aboriginal people think that Aboriginal people are a problem and they kind of pathologise it as if Aboriginality in itself is going to give you worse health and they blame Aboriginal people for that and say things like* “*well*, *if only they*’*d come in here*” *or or* “*if only they*’*d eat properly*.” *And it*’*s with such judgement that it*’*s frightening and that*’*s one of the reasons why I will do everything I can to make sure that cultural awareness training*, *including an understanding of White privilege and dominant culture*, *is undertaken by every staff member on the health site. That*’*s what I want and that*’*s what I*’*ve been working towards for some years. It is very*, *very hard to accomplish that but I*’*m not going to stop trying because I don*’*t think people can fundamentally change*, *I don*’*t think non*-*Aboriginal people can change*, *until they*’*re given the information*. (*HP22*)

Others highlighted that not providing cultural training was a barrier.

Participants identified ***that inclusion of Aboriginal health workers in healthcare teams*** by their organisation was crucial to their success in working with the Aboriginal community. These workers named a key contact person within an Aboriginal health team that they worked with and described how their presence created more opportunity to work in partnership. One worker discussed how it was not appropriate for her to undertake some activities, such as chair a committee to organise National Aborigines (sic) and Islanders Day Observance Committee (NAIDOC) week activities, as she is not an Aboriginal person, and therefore working in partnership with Aboriginal people was vital for good practice.

Health professionals discussed several levels of ***tokenism*** they perceived to exist in mainstream organisations that can negatively influence a health professional’s or an organisation’s capacity to work well with Aboriginal community members. Interviewees identified that organisational policies regarding cultural respect and accountability are tokenistic when they are not reflected in practice. This included Aboriginal health events such as cultural training happening once-off and in isolation and mainstream policies not adequately addressing or not being implemented to meet the cultural needs of Aboriginal employees, making it difficult for Aboriginal people to continue working and meet their cultural obligations. Participants commented that an organisation’s approach to Aboriginal health could appear tokenistic if higher level management appeared to be *very pro*, ‘*let*’*s put Aboriginal health first*, *let*’*s make it a priority*’ (HP17) but tended to have limited understanding of the practicalities of working in Aboriginal health. This often led to unrealistic expectations of timeframes. One participant provided an example of tokenism:

*At* [*community event*], *for example*, *the* [*Aboriginal health*] *manager there was quite critical that they saw that it was tokenistic because the director* [*of the health service*] *only rocked up for like 45 minutes and they are very vocal usually about the fact that Aboriginal health is so important at* [*location*] *but they were only there for 45 minutes for a whole day event*, ***missed the whole Welcome to Country and stuff*** (*emphasis added*) *which is very important*. (*HP12*)

***Staff turnover*** was identified as a barrier to good experiences at the interface, particularly in rural and remote areas where this is common. Some dietitians reported a sense of *mistrust and resistance* being created between non-Aboriginal health professionals within the health service and Aboriginal workers/community due to repeated staff changes:

*Aboriginal Communities have had different non*-*indigenous people coming in over the years and not trusting what they are actually about and having no time to build that trust*. (*HP14*)

A high turnover of Aboriginal health staff was also identified as difficult because it can disrupt established relationships between Aboriginal and non-Aboriginal health professionals.

### Organisational support

Support within an organisation for both Aboriginal and non-Aboriginal staff was considered vital in order for the two groups to work together effectively at the interface.

A ***supportive manager*** was perceived as crucial for success. Dietitians consistently referred to their organisation and management as elements that determined whether or not they were successful in working at the interface. They identified that unless they had a supportive manager who was aware of the issues and challenges of working with Aboriginal communities, they would struggle to do the work. For example, one dietitian talked about her manager who was aware that relationship building was a necessary precursor to achieving health outcomes, and encouraged the use of a community development approach. In contrast, another dietitian did not feel supported by her manager and was not able to be flexible in her approach, which was reflected in outcomes such as *there will be days when you go and you will see two people* (HP10).

### Accessibility of health services

Interviewees identified that whether or not a health service was ***accessible*** to the Aboriginal community affected how well they could work with Aboriginal people at the interface. Having ***welcoming physical spaces*** facilitated connection with the local community and was seen as a way for an organisation to demonstrate that it values its Aboriginal workers and community members. The services accessed by Aboriginal people at one health service were where ‘*the Aboriginal community have a sense of community ownership over the services run largely by Aboriginal people completely for Aboriginal people*’ (HP34). In contrast, another participant talked about how the health service in a rural area was not utilised by Aboriginal people because they did not feel comfortable using it, and therefore there were no interactions between health professionals and Aboriginal people in the health service setting.

### Responding to expectations of the wider health system

Interviewees identified that the organisation in which they worked was part of the ***wider mainstream health system***, meaning that organisations were required to respond to wider health system expectations. For example, there are certain ‘*objectives that we need to meet*’ (HP12), which can limit the ability to be flexible at the organisational level. It was acknowledged that the different approaches and expectations between mainstream and Aboriginal health organisations could make working together difficult, for example:

*Our health system isn*’*t almost set up to be able to have that flexibility with Aboriginal health in general*….***we***’***re trying to get them to fit into our world***, ***we***’***re not adjusting to fit into their world***…(*emphasis added*) (*HP23*)

Similarly, participants were concerned about the measure against which their ***practice is assessed***. It was acknowledged that much of the effort and time required to work well with Aboriginal people at the interface, such as building relationships, is not reflected or recordable in current statistical accounts, which are used to measure services provided within Government health services. It was also identified that within the mainstream health system there may be a ***lack of coordination of services*** related to Aboriginal health, which can lead to programs repeatedly approaching prominent, visible Aboriginal people in the community (such as Aboriginal health teams) with multiple requests. It was suggested that to overcome this, there needs to be ‘*a focus of working with Aboriginal people as core business in all settings*’ (HP23) in ‘*health related disciplines and education and finance and right across the board*’ (HP34).

### The individual

Two main areas were identified within individual health professionals that influenced their experience of working at the interface, personal ideology and awareness of colonisation.

### Personal ideology

Ideology has been described as a set of ideas or a way of looking at things [21] and personal ideologies were identified by interviewees that influenced their experience of working with Aboriginal people at the interface (Fig. 1). Specifically, past experiences shaped health professionals personal ideology which then facilitated or impeded more positive work interactions with Aboriginal people.

Participants referred to a number of ***positive past interactions*** that contributed to their ability to work more positively with Aboriginal people and how prior experiences working with Aboriginal communities helped to provide ‘*an idea about what you are able to do within the program and understand a bit more about the culture and the history*’ (HP7). One dietitian talked about growing up in the Northern Territory, around Aboriginal people. She believed that this made it easier for her to work in Aboriginal health because it was ‘*not that foreign*’ (HP4). On the other hand, ***negative past interactions*** with or about Aboriginal people impacted negatively for two dietitians working in Aboriginal health. They discussed growing up in rural Australia and being exposed to negative beliefs about Aboriginal people, which left them with implicit biases and a sense of fear about working at the interface, with the second worker describing how the fear she held as a child towards the Aboriginal community sometimes resurfaced:

*I grew up*, *and can tell a personal story*, *in Darwin*, *and in walking to school I would walk past members of the Aboriginal community who were inebriated and would ask me for money and harass me if I didn*’*t have it and I was fearful. As an adult*, *more recently I experienced a group of Aboriginal people in Coober Pedy* [*remote community*] *and I was fearful again. Even all the thinking that I*’*ve done*, *I can still flip into feeling frightened and then the kind of shame that went along with that*, *how easy it is to feel unsafe in relationships and how vulnerable we are as people* (*HP29*).

In comparison, three participants identified that having ***come from non***-***dominant cultural backgrounds*** and experiencing hardship in their home countries actually enhanced their ability to empathise with Aboriginal people. One worker talked about how this affected her work:

*Do you know what*, *I think for me it*’*s slightly different because I*’*m a migrant. In my eyes Aboriginal people are the true owners of this land so the way I see things is so different to how White people see things but I can only talk from my own personal perspective*…[]…*I believe that being brown has actually been an advantage for me working with Aboriginal community because I*’*m not of a dominant culture*, *I have a huge respect for Aboriginal community because they have been through a lot. This is their land*, *I*’*m a visitor in their land and so I respect them*, *I respect their culture*. (*HP27*)

For a third group of participants, a lack of any past experience of ***entering a different cultural space*** was identified as making it more difficult to work at the interface. For example, entering another cultural space was described as taking a risk, where a health professional is required to step out of their comfort zone, work in a situation where they do not feel confident and be in a physical place where they are likely to be in the minority, which can cause discomfort.

Health professionals with the most experience working in Aboriginal health (more than 15 years, *n* = 7) engaged in ***deep self***-***reflection*** about their work in Aboriginal health. This involved a deep, detailed consideration of themselves including their own position, stereotypes, biases and relationship to the Aboriginal people they worked with. These health professionals were able to identify and name their implicit biases. Emerging from such reflections was awareness that ‘…*firstly you have to admit that you don*’*t know*’ (HP12) and that there is a lot more to learn. One worker described this as:

*It*’*s a Johari*’*s Window*, *you know that thing about you know what you know and then there is this part where you can*’*t know what you don*’*t know*, *but sometimes you intuitively sense it and you think* “*I am not getting this quite right*” *and sometimes*, *if you*’*re lucky*, *you*’*ll have somebody or people to work with who will say* “*if you*’*d done that in that order*” *or* “*what you forgot to do was that*”, *it moves me along*. (*HP22*)

Closely related to self-reflection was the discussion by non-Aboriginal workers about the ***realities and challenges of working in Aboriginal health***, for themselves and their Aboriginal colleagues. One worker talked about the discomfort he felt working at the interface and that even though he enjoyed the work, ‘*you*’*ve got to push yourself every day to do it*’ (HP12). Another experienced worker posed the question ‘*how do you remain hopeful in the face of so much chaos and despair*?’ (HP29). Similarly, one worker talked about ***racism*** as a daily reality that many Aboriginal people have to face, and this awareness influenced how she worked with Aboriginal colleagues and clients:

*I think that getting up every morning and knowing that you might face racism today* - *I think that deeply affects your health* …[]…*The other thing that I think is a great difference in an Aboriginal person*’*s health is their cultural safety and that sense of* – *people from the dominant culture have a sense of that we have a right*, *we have an unalienable right to go wherever we like to do pretty much whatever we like within the* [*White*] *law*, *that we have a blessing to move freely and to receive services equally with everyone else. I think that for Aboriginal people*, …..[*they*] *don*’*t necessarily feel that they*’*re going to get equal treatment*, *fair treatment*, *social justice wherever they move*, *so whether you*’*re dealing with the local deli or dealing with trying to get housing or whether you*’*re trying to set up a business and want to borrow some money from a bank*, *it*’*s not the same*. (*HP22*)

The impact of various levels of ***confidence*** became apparent. Participants commonly reported a lack of confidence in working at the interface. Their reasons included feelings of frustration, a lack of training, being overwhelmed and having a sense of not knowing what they do not know. Two dietitians reported feeling confident when they started working in this area, but then this level of confidence was challenged. One described initially approaching his work with Aboriginal people with ‘*this dietitian missionary zeal wanting to save the world*’ (HP20). However, this did not last long, and as described by another dietitian ‘*when you start out you have optimism and you think you know it all and then the more you work in the area you find out that you don*’*t know anything*’ (HP11), suggesting that over-confidence quickly makes way for a lack of confidence. For some dietitians, an initial lack of confidence eventually led to an acceptance that they were never going to get things totally ‘right’ when at the interface. Overall it was found that confidence levels changed over time; and even those with more experience reported going through periods and stages when they questioned their work and their abilities to work in this area.

There was also a sense among interviewees that working at the interface was often complicated, difficult and uncertain. Many workers were ***fearful***, and one questioned where this fear came from, concluding that the origins of the fear ‘*is more from the practitioner than it is from the community*’ or that ‘*sometimes the system*, *the bureaucracy*, *the organisation can install a sense of fear or try to tell you that you don*’*t know enough* (HP12).

This fear at times became quite disabling, as this worker describes:

*Well sometimes all the evidence is like* “*you*’*ve got to do this and you*’*ve got to do that*” *and you almost get absorbed in that and start believing in that. You forget that* “*hey it is just another person*” *and they just want you to be genuine and they just want you to be considerate and you just want to work together. You just get hung up on these things and then become hesitant about approaching them and the whole almost becomes a barrier for you to reverse that*. (*HP12*)

There was also fear of doing something wrong, and a perception amongst those with less experience that there are certain skills or knowledge you ‘have to know’ to begin working in this area.

[*There is the belief amongst dietitians that*]….*there are a lot of cultural norms and cultural nuances that you are sort of never going to get right so again there is that stigma attached*, “*well it*’*s all too hard because I don*’*t want to sort of go offending anybody or go making any wrong moves or inappropriate gestures or inappropriate language*”. (*HP34*)

For this participant, this was related to a fear of not fully understanding Aboriginal culture.

### Awareness of colonisation

Interviewees had different levels of understanding of past and ongoing colonisation practices and this impacted on how they viewed and worked with Aboriginal people at the interface. Generally they explained their understanding of colonisation by relating it back to a ***story or a personal connection*** with an Aboriginal person they had worked with and others referred to the Stolen Generation.

In contrast to workers who had reached a level of understanding of colonisation, other workers ***lacked even a basic understanding***. Two dietitians were unsure what the term ‘colonisation’ referred to. Some understood the question and acknowledged that colonisation must still have some impact on the lives of Aboriginal people today but felt somewhat overwhelmed by, for example ‘*all the history out there that you can*’*t get your head around*’ (HP10). Others tried to relate it to their practice but could not see how it fit:

…*I suppose that there are actually quite some tensions* [*in the workforce*] *at the moment I think and I don*’*t know how that sort of fits into colonisation*. (*HP13*)

One participant quickly identified that he felt he ***did not understand colonisation***:

*I*’*d be absolutely lying if I thought I understood it* [*colonisation*] *because the longer I work there the longer I realise I don*’*t know*…[]…*I haven*’*t worked in the area long enough to understand but it is definitely an issue*, *it*’*s huge*…[]…*I am not an expert and I*’*m probably too uncomfortable to comment on it but all I know now is that I have more questions*. (*HP12*)

However, despite this lack of understanding, he was clearly interested and willing to find out more.

While it was generally acknowledged by interviewees that colonisation must continue to impact on Aboriginal people today, most were uncertain of what this would look like in practice in terms of how it would affect their work at the interface. Only interviewees with more experience or self-reflection reported incidents of trauma and negative experience, disempowerment, and generational grief. One experienced worker suggested that a lack of knowledge of history can mean people are not aware of how the past continues to affect the present:

*Basically we are not very good about knowing our own history. Aboriginal people know theirs extremely well. You know*, [*people ask*] “*why did we have to say sorry*, *why do they keep going on about it*?” *And whilst we acknowledge we need to move forward*, *we can*’*t forget the past. I*’*ve got a heap of mates who are always sort of on about that*, *you know*, *and so there is just that lack of understanding. So people need to hear the stories and stuff to understand the big things that impact on communities*, *that*’*s crucial*. (*HP3*)

Other ways that workers identified that ***colonisation could continue to affect the lives of Aboriginal people today*** were through the Stolen Generation, the practice of White workers, storytelling (passing experiences between generations), intergenerational effects, health issues, responses of Aboriginal people and continued racism. One worker used an analogy of an iceberg to explain his understanding of how colonisation continues to effect Aboriginal people today.

…*the analogy of an iceberg*, *90*% *under the water and unseen*, *so often when I*’*m working with Aboriginal men I*’*ll draw this iceberg on a whiteboard and we*’*ll talk about the presenting issues and what people see*, *especially the White community*, *you know*, *violence*, *grog*, *drugs*, *family breakdown*, *mental illness*…. *But what often people don*’*t see is like an iceberg*, *under the waterline*….*we can go back to colonisation or invasion and all the losses that went with that* – *loss of land and language and ceremony and culture and identity* – *all the murder*, *rape*, *slavery all that stuff that is often not talked about*, *abuse of power by a White dominant culture and then Aboriginal men involved in all major wars*, *all that historical stuff*. (*HP9*)

It was also acknowledged that non-Aboriginal workers could perpetuate colonisation through their practice at the interface and beyond; through using a paternalistic approach and appearing to have all the answers when working with an Aboriginal person; or through a lack of recognition of history and the hardships faced by Aboriginal people.

Three participants with more than 15 years’ experience each in Aboriginal health reflected on the idea that the impact of colonisation and associated events is ***intergenerational***. One worker described this as:

*I don*’*t think we could overestimate how much colonisation*, *invasion*, *disrespect*, *illegal acts*, *it*’*s immeasurable how much damage that*’*s done and if you damage my grandmother*, *if you damage my mother you damage me*, *you know. It is like that damage*, *that hurt*, *you carry through*. (*HP22*)

Another reflected that Aboriginal people living on missions were not allowed to make decisions about their lives, and how this is passed from one generation to the next and consequently impacts on Aboriginal people today and therefore on interactions with health professionals at the interface. A third discussed the impact of these intergenerational effects, for example carrying around grief that is part of the individual and their history, which complicates things in everyday life such as work.

Those interviewees with a greater understanding of colonisation and its impacts discussed how they change the way they work with an Aboriginal person to take account of this. Their strategies were based around ***approach***, ***communication***, ***organisation and individual action***.

These participants discussed using flexible and responsive ***approaches*** to practice that did not reinforce colonisation. One worker talked about sitting back, waiting, going slowly and waiting for Aboriginal people to come to you and another discussed compromise:

…*rather than making them do things our way you*’*ve got to ring this number and then you*’*ve got to wait 3 weeks and then you have to do xyz*, *we*’*ll try and meet you at least half*-*way*. (*HP19*)

In terms of ***communication***, workers highlighted the importance of making connection through asking people where they are from, and showing interest and sharing stories. It was acknowledged that each Aboriginal person has their own, individual story to tell. Some shared stories about themselves and saw this as an important part of building rapport:

*One day they might ask me about my story and background and that*’*s when you know that things are a little bit easier*. (*HP21*)

At the ***organisational level***, experienced workers discussed that making access to health services as easy as possible for Aboriginal people was one way to acknowledge and counter the effects of colonisation on Aboriginal people. Similarly, ‘*support* [*ing*] *Aboriginal Health Workers in doing their job in whatever way that might be*’ (HP2) and acknowledging the impact of colonisation and its continued effects through a formal organisational document in order to ‘*make that really clear that we know that is a fact*’ (HP29).

Strategies that ***individuals*** used to address colonisation in their practice at the interface were also discussed. Being aware of Aboriginal history and making an active effort to learn about it was seen as important, in particular the history of local Aboriginal people. The value of being aware of issues facing contemporary Aboriginal people, particularly through watching films and television programs about contemporary Aboriginal issues, visiting Aboriginal camps, learning local language and using it and learning from Aboriginal people, was highlighted. Finally, being open to learning from Aboriginal people and acknowledging the impact that colonisation has had on the lives of Aboriginal people were seen to be important.

## Discussion

This paper presents the experiences of non-Aboriginal health professionals (primarily allied health professionals) working at the interface in Aboriginal health. It demonstrates that there are factors which facilitate or constrain a health professional to effectively and skilfully work at the interface. These fell into two key areas – the organisation and the individual - within which barriers and enablers were identified. This paper highlights that work at the interface cannot be considered in isolation from the individual/personal and organisational context in which a health professional works.

Organisational management and the wider system within which that organisation operates have previously been shown to influence the experience of health professionals working in Aboriginal health. For example, organisational capacity and barriers directly impacted on effectiveness and delivery of interventions related to smoking, nutrition, alcohol and physical activity to Aboriginal clients [27]. Providing a supportive physical and virtual space to allow partnership development between Aboriginal and non-Aboriginal people is required for development of partnerships, for example in a program seeking to cease petrol sniffing and another about aged care services [28, 29]. Further supporting the work reported in this paper, staff turnover has previously been identified as an organisational barrier because it erodes trust between mainstream health services and Aboriginal community members, prevents the development of trusting relationships between Aboriginal and non-Aboriginal workers and impacts on the delivery of interventions, while a supportive mainstream health service manager is paramount to the delivery of successful health care to Aboriginal clients [27, 30].

Past experiences and interactions with Aboriginal people shaped the personal ideology of health professionals which assisted or hindered positive healthcare interactions with Aboriginal people. Personal ideology is an important concept because it returns some agency to the health professional practising at the interface. For example, if health professionals seek more positive healthcare interactions with Aboriginal people at the interface, even in the presence of organisational barriers, they can focus on addressing personal ideology. The importance of health professionals self-reflecting, evaluating and increasing their self-awareness both personally and professionally has previously been acknowledged, for example a critique of one’s own practice [31], reflection on one’s beliefs, attitudes, values and worldviews [32–35], awareness of assumptions [33], knowledge of one’s limitations [36], preconceived ideas and stereotypes [37, 38] and motivation to work with Indigenous peoples [32] Health professional attitudes directly affect practice in Aboriginal health, for example physicians’ attitudes towards Aboriginal people affected the care they provided [39], demonstrating the importance of personal ideology as a contributor to experience. The presence of implicit biases about Aboriginal people, developed from past interactions, were also evident in health professionals, with some able to mitigate the negative effects of these when working at the interface, generally through self-reflection. Previous research has identified that the implicit bias of health professionals leads to disparities in health. For example, there is a body of literature from the USA indicating that health professionals hold unconscious beliefs about people from different minority populations [40, 41] and that these beliefs affect their treatment decisions which ultimately affects patient health [42, 43]. Further research is required in relation to the role of implicit bias amongst health professionals on the health outcomes of Aboriginal Australian people. However, research in Australia has indicated that individual racism contributes to poor health of Indigenous Australians, and it is highlighted that there needs to be fundamental changes to how non-Aboriginal people behave towards Aboriginal people in order to address health inequalities-improved health care and other initiatives are not enough [4].

Given the lack of understanding of the concept of colonisation by some health professionals in this research, health professional training must teach concepts of colonisation and decolonisation. In teaching health professionals, it is important to move from a colonising, deficit-based approach which constructs Aboriginal people as the ‘problem’ to a decolonising, Indigenous rights and strengths-based approach which ensures that, for example, colonisation, invasion and removal of Aboriginal peoples’ sovereign rights provide a context for health status which must be acknowledged and that Aboriginal knowledges are recognised, valued and privileged in relation to Aboriginal health [44]. Clearly, this is a task that requires sustained implementation over a long period of time in Australia and at multiple levels (individual, organisation, health system). However, it has been argued that unless colonisation and the continuation of a colonising agenda today is acknowledged and steps are taken towards decolonisation, Aboriginal health cannot be effectively addressed [45]. In support of this, previous research has demonstrated that having an awareness of and knowledge about Aboriginal history and culture is important [33, 37, 46–48] and historical events impact and shape relationships between practitioners and Aboriginal communities in present day Australia [49].

A critical step towards decolonisation is a personal critique and reflection on the common, White constructions of history that informs assumptions and perceptions [45] and this must be included in health professional teaching and professional development. However, systems in Australia (including the health system, education system, organisations and health professional organisations) also need to consider the implicit structures and practices within them that privilege White paradigms and perpetuate a colonising agenda. Unless this is understood, the notion of decolonisation cannot be grasped and actioned by individuals, organisations and systems. Health professional training must focus on personal critique and reflection [45] including a recognition of whiteness, its unmarked and unnamed status [50] and the privilege that this confers upon members of the dominant culture in Australia [51]. Decolonisation involves acknowledgement and comprehension by all Australians the impact that invasion, imperialism, colonisation, removal of Indigenous rights, research and policy have had on Australian Indigenous peoples [44]. There was evidence of decolonising practice by some health professionals in this research, for example the use of approach, communication, organisation and individual action to not reinforce colonisation. Further research in health organisations and with health professionals is required to assess the extent to which an awareness of colonisation and decolonisation exist at the organisation and health systems level exist, and whether health professionals, organisations and the health system consider this to be important.

It is clear from this work that many factors affect the experience of non-Aboriginal health professionals working at the interface in Aboriginal health. This is a positive finding because it provides greater indication of areas that can be addressed to train health professionals to work with Aboriginal people and ultimately address health equity. For example, the two main areas (individual and organisation) provide options for where practitioners and managers might look to make changes/develop. However, the importance of both of these areas also highlights that even when a health professional has a lot of positive individual factors that enable them to work at the interface, they may still struggle or have a negative experience due to organisational factors. A factor that influences a health professional’s experience at the interface may lead to an overall positive or negative experience at the interface depending on how it is experienced and the combination of factors at play. As an element of professional development, health professionals could be encouraged to think about which area they can focus on at this point in time, and those in management roles could consider organisational elements which may require addressing.

## Conclusion

Work at the interface cannot be considered in isolation from individual and organisational contexts. This study provides some insight into why many health professionals find Aboriginal and Torres Strait Islander health a complex area to navigate and work, despite previous work on what constitutes good practice in this area [17, 52]. The model presented provides some good starting points for areas to address in health professional training and professional development. It also acknowledges that there are some deep issues that need careful consideration how to address and how to best up skill health professionals to manage, for example an understanding of colonisation. Further work is required to explore what factors influence the patient’s experience of receiving care at the interface and whether or not the type of care a patient receives at the interface affects their health outcomes. At this point, it is critical to consider how the organisational and individual factors identified in this research will be addressed in health professional training and practice, in order to maximise the ability of health professionals to work with Aboriginal and Torres Strait Islander people, and thus address health equity.

## Abbreviation

NHMRC: National Health and Medical Research Council

## Footnotes

1
*Hereafter referred to as Aboriginal*: *The term* ‘*Aboriginal*’ *is used in this context to be inclusive of Aboriginal and Torres Strait Islander peoples. This use of terminology is considered preferable to the term* ‘*Indigenous*’ *and is consistent with the position statement of the National Aboriginal Community Controlled Health Organisation. We use the term* ‘*Indigenous*’ *and* ‘*Aboriginal and Torres Strait Islander*’ *in this paper when these words are part of a title or name or when citing work that refers specifically to these terms*.
